# A scoping review of health promotion interventions delivered via social media to women of reproductive age

**DOI:** 10.1017/S136898002300246X

**Published:** 2023-12

**Authors:** Maddison J Henderson, Megan L Gow

**Affiliations:** 1 The Children’s Hospital Westmead Clinical School, The University of Sydney, Westmead, NSW 2145, Australia; 2 Discipline of Paediatrics and Child Health, School of Clinical Medicine, University of New South Wales, Sydney, NSW, Australia; 3 Women’s and Children’s Health, St George Hospital, Kogarah, NSW, Australia

**Keywords:** Women’s health, Health promotion, Social media, Mental health, Physical health

## Abstract

**Objective:**

Adult women of reproductive age are highly engaged with social media, suggesting its utility for conveying health information to this population, at scale. This scoping review aimed to describe health promotion interventions conducted via social media and assess their effectiveness to improve health outcomes, engagement and acceptability in adult women of reproductive age.

**Design:**

Six databases were searched on 13 May 2022. Two reviewers independently screened studies. Data were extracted and risk of bias assessed using the Joanna Briggs Critical Appraisal Tools.

**Setting:**

Eligible studies conducted an intervention primarily via social media, with or without a comparison intervention, and reported health-related outcomes/behaviours pre- and post-intervention. Results were presented in narrative form.

**Participants:**

Adult women (mean age 18–45 years).

**Results:**

Nine eligible studies were identified: six randomised control trials, two quasi-experimental studies and one cross-sectional study. Interventions focused on prenatal, antenatal or postpartum health or physical activity. Seven studies utilised Facebook for intervention delivery, one conducted a digital campaign across four platforms and one used WeChat. Studies reported significant improvements in a range of outcomes, including increased parenting competence, longer duration of breast-feeding and higher rates of physical activity. Social media interventions had greater engagement than control interventions.

**Conclusions:**

We identified nine diverse health promotion interventions conducted via social media, which appear acceptable and effective for improving various health outcomes in adult women of reproductive age. While this supports the utility of social media to convey health information, further research is required to prove effectiveness and superiority over other intervention strategies.

The term ‘social media’ broadly refers to any website or application that allows its users to generate and exchange content^(1)^. In recent years, the number of active social media users worldwide has continued to increase, rising from 2·73 billion in 2017 to 4·59 billion in 2022^(2)^. By 2027, this number is projected to reach 5·85 billion.

In particular, adult women of reproductive age are highly engaged users of social media. Globally, 35 % of Facebook’s and 40 % of Instagram’s current users are females aged between 18 and 54 years^(3,4)^. Across all age groups, the highest proportion of Facebook’s and Instagram’s female users was aged 25–34 years, representing 13 % and 15 % of users, respectively.

As a result of their global accessibility and scalability, social media platforms are emerging as a favourable tool in health research. This includes health promotion interventions that aim to promote changes in health and health behaviours, such as physical activity levels, diet quality, anthropometric measurements and psychological health^(5)^. In addition, online interventions can easily be accessed by participants on a range of devices, including computers, laptops, mobile phones and tablets, at their convenience. These interventions may also be more cost effective than traditional interventions^(6,7)^.

This ease of access could be particularly advantageous for women of reproductive age, especially those with infant children, as it may allow a population with competing family, work and personal demands to participate in health promotion interventions at their convenience while eliminating the need to attend time-consuming in-person sessions^(6,8)^. However, little is known about how health promotion interventions conducted via social media affect the health and health behaviours of women of reproductive age, or whether this is an acceptable platform for intervention delivery. To date, no review has provided a synthesis of the field to describe health promotion interventions conducted via social media and examine their effect on health-related outcomes and behaviours of women of reproductive age. If these types of interventions are shown to be acceptable and effective for improving women’s health, further research in this area would be supported, including the design of health promotion interventions to be delivered at-scale via social media to improve population health.

Therefore, this scoping review aimed to primarily describe health promotion interventions delivered to women of reproductive age via social media. Further, this review aimed to determine whether the health promotion interventions have a positive effect on the mental or physical health or health-related behaviours, including diet and exercise, of adult women of reproductive age and determine the level of engagement with, and acceptability of, the intervention by women.

## Materials and methods

The Preferred Reporting Items for Systematic Reviews and Meta-Analyses Extension for Scoping Reviews (PRISMA-ScR)^(9)^ was used to guide this systematic review. Additionally, the review was registered in the International Prospective Register of Systematic Reviews on 8 June 2022 and was assigned the study protocol registration number 336812.

### Search strategy for study identification

Six electronic databases were systematically searched on 13th May 2022 by one author (MH) to identify relevant studies: MEDLINE, Embase, PsycINFO, CINAHL Complete, Cochrane Library and Scopus. Advanced searches in titles, abstracts and keywords were conducted using a combination of population terms (e.g. postpartum women, pregnant women), intervention terms (e.g. health promotion, social media, internet-based intervention) and outcome terms (e.g. mental health, depression, anxiety, physical activity, diet). To ensure a comprehensive search of the literature, no restrictions were placed on comparison interventions or year of publication. Search strategies were adjusted for each database (online Supplemental Table 1). Reference lists of retrieved papers were also reviewed to identify relevant publications for inclusion.

### Eligibility criteria

Studies were eligible for inclusion in the present review if they (1) recruited adult women of reproductive age (mean age 18–45 years)^(10)^, including pregnant and postpartum women; (2) delivered a health promotion intervention primarily via social media; (3) assessed and reported at least one health-related outcome (e.g. mental health, weight, blood pressure) or behaviour (e.g. knowledge, awareness, diet, physical activity, infant feeding practices) pre- and post-intervention; (4) were written in English and (5) were accessible in full text. Studies were excluded if they (1) recruited mixed populations (e.g. adult males or adolescent females in combination with adult women) and did not disaggregate data for adult women; (2) only used social media for recruitment purposes; (3) delivered an intervention primarily face-to-face, or via mobile phone applications (not including mobile versions of social media platforms), websites, text messages, phone calls and/or emails; (4) only used social media as a forum for support during the intervention period; (5) did not report any health-related outcomes or behaviours or (6) were a systematic review, meta-analysis, study protocol or conference abstract.

### Selection of studies

The study selection process was undertaken using Covidence Systematic Review Software. Studies identified by the six electronic databases were exported into Covidence and duplicates were automatically identified and removed. Both authors independently screened the titles and abstracts of all studies and irrelevant articles were excluded. The remaining studies progressed to full-text screening and were further assessed against eligibility criteria. This stage was also conducted in duplicate. Discrepancies throughout the study selection process were discussed and resolved by consensus between the two authors.

### Quality assessment

Although not a requirement of a scoping review, a risk of bias assessment was completed using the Joanna Briggs Institute Critical Appraisal Tools. The corresponding checklist was utilised for each study design as appropriate^(11,12)^. For each item, possible answers included ‘yes’, ‘no’, ‘unclear’ or ‘not applicable’. Studies were classified as low risk of bias if they scored ‘yes’ for at least 70 % of items, moderate risk if they scored between 50 and 69 % and high risk if they scored less than 50 %^(13)^. Both authors independently utilised the relevant checklist for each study type to determine the risk of bias for the included studies. Discrepancies were discussed and resolved by consensus.

### Data extraction and synthesis

A data extraction form was developed in Microsoft Excel and relevant study characteristics were extracted by one author (MH). This included publication information (first author, year of publication, study location), methodological details (study design, sample size), participant demographics (age, gender, pregnancy status, ethnicity/socio-economic status), intervention details (social media group, comparator group, duration, retention, data collection methods) and study outcomes (main findings for health outcomes or behaviours, findings regarding the success of the social media intervention, i.e., engagement or acceptability). The completed data extraction form was reviewed for accuracy and completeness by the other author (MG). Given the limited number of studies included in the present review and the heterogeneity of these studies, the results were synthesised and presented in narrative form.

## Results

### Study selection

**Fig. 1:**
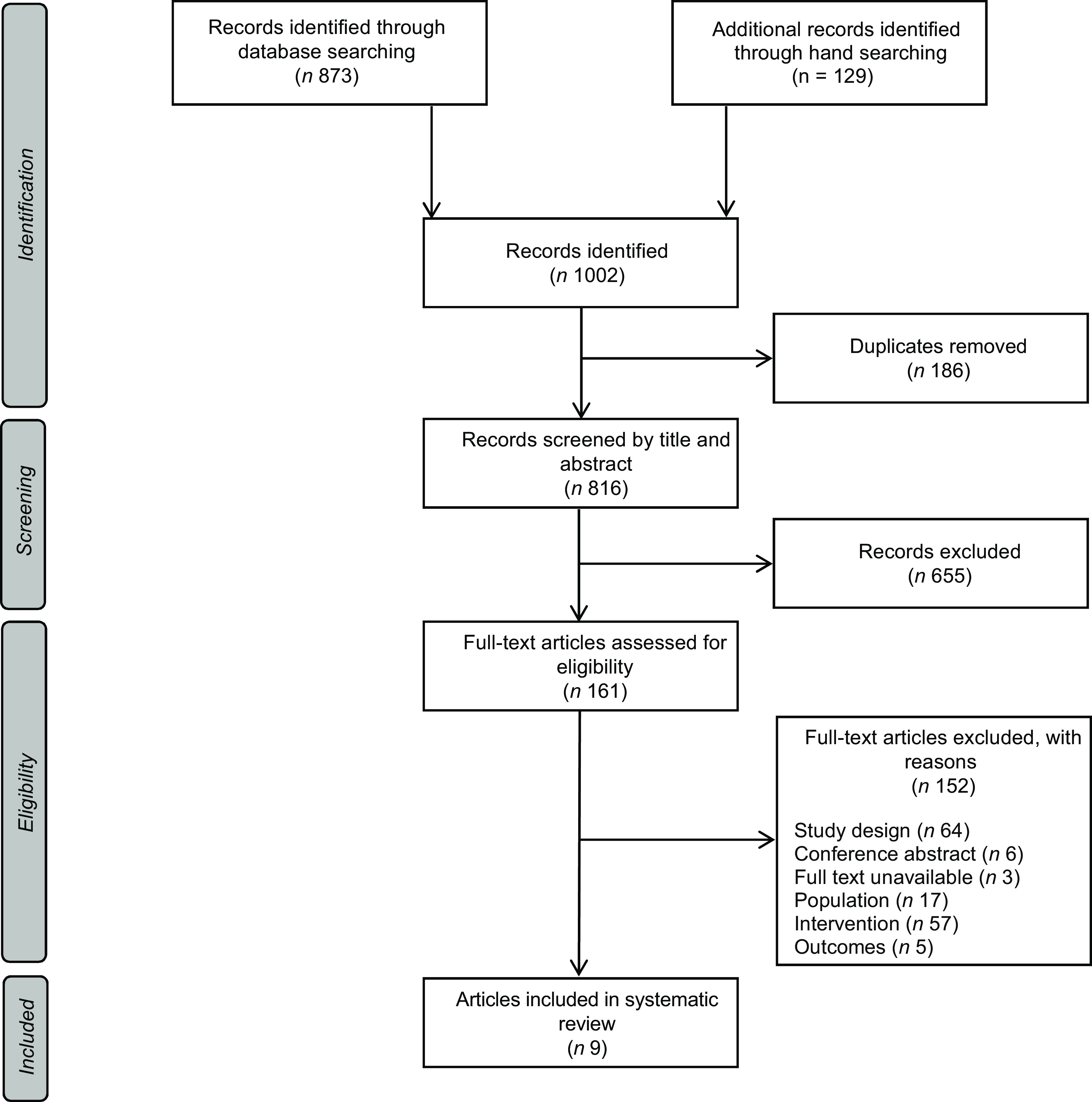
PRISMA flow diagram

The combined database searches produced a total of 873 records and 129 additional records were identified from reference lists of retrieved articles (Fig. 1). Following the screening process, nine studies were eligible for inclusion in this review.

### Study characteristics

#### Study design

The characteristics of the included studies are presented in Table 1. Study designs included randomised control trial^(14–19)^, quasi-experimental^(20,21)^ and cross-sectional^(22)^. Most studies (*n* = 6) were conducted in the USA^(14,16–18,20,22)^, with the remaining studies conducted in Brazil^(15)^, Taiwan^(21)^ and China^(19)^. The health domains assessed were prenatal health^(22)^, antenatal health^(19–21)^, postpartum health^(14–16)^ and physical activity^(17,18)^. Studies were published between 2015 and 2021. Most interventions (*n* = 8) were conducted for less than 1 year (i.e. between 2 and 11 months)^(14–21)^ and the remaining study intervention was implemented for 2 years^(22)^. No studies provided follow-up beyond the intervention period. Seven studies included a control group^(14–19,21)^, such as standard care or a face-to-face intervention.

**Table 1:** Characteristics of the included studies

			Participants									
Author, year (country)	Study design	Health domain	Total (IG; CG)		Demographics	M age IG; CG (yrs)	Social media intervention	Comparator	Duration	% Retention(IG;CG)		Data collection method; health outcomes and behaviours
Bonnevie, 2021 (USA)^(22)^	CS	PN	176 – BL: 30, Yr1: 61, Yr2: 85		18–65 yrs, female, self-identified as Black, lived in 1 of 15 zip codes in O.C.	N/R (majority <45)	Digital campaign (*Strong Beautiful Future*) that provided pregnancy-related information via social media (Instagram, Facebook, Twitter and YouTube) and a website. Content was posted 5–7 times/wk	N/A	2 yrs	N/A		Online surveys at BL, Yr1 and Yr2 (designed by authors); PN care behaviours, attitudes towards specific health behaviours during pregnancy, and knowledge about LBW and its impacts on the fetus or infant
Boyd, 2019 (USA)^(14)^	Pilot RCT	PP	24	12; 12	>15 yrs, mothers with a child aged 1-3 mths, internet access on computer or phone, screened positive for depressive symptoms	26·4; 26·3	Parenting intervention (*PIWI*) delivered via a private Facebook group. A new topic was covered each wk in 3 instalments	*PIWI* delivered in person. Sessions were held wkly	8 wks	84	100; 67	Surveys at BL and post-intervention: BDI-II; severity of depression symptoms PSOC; perceptions of parental competence
Cavalcanti, 2019 (Brazil)^(15)^	RCT	PP	251	123; 128	> 18 yrs, mothers who gave birth between Aug 2016 and Feb 2017, literate, Facebook user	N/R (Mdn 26; 27)	Information booklet on the practice of breast-feeding and inclusion in a private Facebook group (*Mama Breast-feeding Project*). Once a wk, the mothers were tagged in a post, which corresponded to a topic of the information booklet	Information booklet only	6 mths	97	94; 99	Mthly phone calls; EBF
Fiks, 2017 (USA)^(16)^	RCT	PP	85	43; 42	≥ 18 yrs, pre-pregnancy BMI ≥ 25 kg/m^2^, Medicaid insured, English speaking, singleton pregnancy between 20 and 32 wks gestation, owned a smartphone with a data plan that could take photos and videos	25·8; 27·3	Text msg apt reminders and inclusion in a private Facebook group (*Grow2Gether*). Videos relating to healthy parenting and infant growth were posted wkly until infant age 6 mths, then they were posted bi-wkly. Four groups were created, and each contained 9–13 women with similar due dates	Text msg apt reminders only	11 mths	84	79; 88	Surveys at T0 (screening/enrolment), T1 (following birth), T2, T4, T6 and T9 (infant ages 2, 4, 6 and 9 mths): IFSQ; infant feeding practices MSPSS; support from others BISQ; infant sleep patterns HOME; quality of the home environment PSS; feelings about parenting role KPCS; parenting self-efficacy
Joseph, 2015 (USA)^(17)^	Pilot RCT	PA	29	14; 15	24–49 yrs, self-identified as AA, female, insufficiently active (<150 mins/wk moderate PA), active Facebook account, could read and write in English	35·6; 35·3	Culturally relevant PA intervention delivered via a private Facebook group and text msgs. PA promotion materials were posted in the group wkly and motivational text msgs were sent 3 times/wk	Print-based intervention, consisting of 4 non-culturally tailored self-help booklets promoting PA. These were mailed each fortnight	8 wks	100 (however 1 participant in IG did not provide valid accelerometer wear time and was not included in the analysis)		Surveys at BL and post-intervention: EVS; self-report frequency and duration of PA Exercise Confidence Survey; self-efficacy for PA Social Support for Exercise Survey; social support for PA Self-Regulation Scale; self-regulation for PA OEE; outcome expectations for PA PA at BL and post-intervention: Accelerometer; time spent in sedentary, light, moderate and vigorous PA and daily step counts PA during the intervention: Pedometer and calendar; self-monitoring of total steps and mins of walking/d Anthropometric measures at BL and post-intervention: Ht and Wt (measured by a senior staff member); changes in anthropometric measurements
Rote, 2015 (USA)^(18)^	RCT	PA	63	32; 31	Female, college freshman, lived in an on-campus dormitory, Facebook user, insufficiently active (<7500 steps/d)	18·6 (overall)	Standard walking intervention and inclusion in a private Facebook group. Educational information on PA was posted wkly. The women were also sent a personal Facebook msg with feedback and their new step goal each wk. Four groups were created	Standard walking intervention. Educational information, feedback and step goals were emailed each wk	8 wks	84	84; 84	PA at BL and during the intervention: Pedometer; steps/d (wkly logs were also used during the intervention) Anthropometric measures at BL and post-intervention: Ht, Wt, BMI and WC (measured by research staff); changes in anthropometric measurements
Vander Wyst, 2019 (USA)^(20)^	Pilot QE	AN	12 (IG only)		>19 yrs, pregnant women carrying a single fetus who were between 12 and 28 wks gestation, low-to-medium income	29·2 (IG only)	Health information relating to pregnancy was sent out via Facebook and/or cellular text msgs. Six msgs were sent/wk	N/A	18 wks	100		Pre-pregnancy Wt and hospital delivery Wt; GWG Nutrition knowledge survey (designed by the researchers) at BL and post-intervention; knowledge of basic nutrition concepts 24 h diet recalls at visits closest to 0, 9 and 18 wks; diet quality Interviews at BL and post-intervention; maternal attitudes and beliefs about PN health
Wu, 2019 (Taiwan)^(21)^	QE	AN	121	66; 55	> 20 yrs, pregnant women <12 wks of gestation, > 9 yrs of education, no complications or underlying medical problems during the pregnancy, access to the internet (IG only)	32·8; 32·8	Ordinary PN care and inclusion in a private Facebook group (*EMC*), which contained a discussion forum and library area for pregnancy-related information	Ordinary PN care	Up until 36–38 wks gestation	90	89; 91	Surveys during each trimester (<12, 22–24 and 36–38 wks): The Symptoms Checklist; physical symptoms EPDS; depression The Social Support Scale; extent of support received from family, peer pregnant women, friends and HCP Maternal Fetal Attachment Assessment Scale; maternal-fetal attachment The Pregnancy Adaptation Scale; pregnancy adaptation
Yang, 2019 (China)^(19)^	RCT	AN	123	62; 61	> 18 yrs, pregnant women between 24–30 wks gestation, low-risk pregnancy, internet access, fluent in Chinese, able to complete the questionnaires, elevated depressive or anxious symptoms	31·3; 30·4	Mindfulness intervention delivered via a private WeChat group. 4 × 40 min mindfulness sessions were uploaded throughout the intervention. The women could also access text, pictures and audio during the intervention	Ordinary PN care and inclusion in a private WeChat group to interact with CG and researchers only	8 wks	83	84; 82	Surveys at BL and post-intervention: PHQ-9; symptoms of depression GAD-7; level of general anxiety FFMQ; level of mindfulness

AN, antenatal; Apt, appointment; BDI-II, Beck Depression Inventory-II; BISQ, brief infant sleep questionnaire; BL, baseline; CG, control group; CS, cross-sectional study; EBF, exclusive breast-feeding; EMC, Expectant Mother Club; EPDS, Edinburgh postnatal depression scale; EVS, exercise vital sign questionnaire; FFMQ, five facets of mindfulness questionnaire; GAD-7, generalised anxiety disorder scale; GWG, gestational weight gain; HCP, health care professional; HOME, home observation for measurement of the environment; Ht, height; IFSQ, infant feeding style questionnaire; IG, intervention group; KPCS, Karitane parenting confidence scale; LBW, low birth weight; M, mean; Mdn, median; Min, minute; Msg, message; MSPSS, maternal scale of perceived social support; Mth, month; N/A, not applicable; N/R, not reported; O.C., Orange County; OEE, outcome expectation scale for exercise; PA, physical activity; PHQ-9, the patient health questionnaire; PIWI, Parents Interacting With Infants; PN, prenatal; PP, postpartum; PSOC, parenting sense of competence scale; PSS, parental stress scale; QE, quasi-experimental study; RCT, randomised control trial; WC, waist circumference; Wk, week; Wt, weight; Yr, year.

#### Study sample

The number of participants in each study ranged from 12 to 251 (mean number of participants = 98). Post-intervention retention rates for all studies except Bonnevie *et al*.^(22)^ ranged from 83 % to 100 % (mean retention = 90 %). Bonnevie *et al*.^(22)^, which had a greater uptake of cross-sectional survey completion as the duration of the intervention went on, had 30, 61 and 85 women complete a cross-sectional survey at baseline, Year 1 and Year 2, respectively. Most studies (*n* = 6) recruited participants from hospitals^(15,19)^, clinics^(14,16,20)^ or medical centres^(21)^. Only one study^(22)^ used advertisements on social media for recruitment.

The mean age of participants in the majority of included studies (*n* = 7) ranged from 18 to 36 years^(14,16–21)^. However, two studies^(15,22)^ did not report a mean age and instead noted that they recruited women aged 18–65 years (majority were under 45 years)^(22)^ or > 18 years (median age was 26 and 27 years in the intervention and control group, respectively)^(15)^. Six studies exclusively recruited pregnant^(16,19–21)^ or postpartum women^(14,15)^. Fiks *et al*.^(16)^ and Vander Wyst *et al*.^(20)^ further restricted their inclusion criteria to low- to medium-income women, Boyd *et al*.^(14)^ to mothers with postpartum depression and Yang *et al*.^(19)^ to women with mild to moderate symptoms of anxiety or depression. Although Bonnevie *et al*.^(22)^ focused on recruiting Black women, 21·1 %, 29·5 % and 20·0 % of participants at baseline, Year 1 and Year 2, respectively, were reportedly pregnant. Six studies reported on participant ethnicity. All or most participants (i.e. >80 %) in four studies were African American/Black^(14,16,17,22)^, whereas the other two studies primarily recruited White women^(18,20)^.

### Intervention characteristics

Eight studies utilised Facebook to deliver their intervention, either in isolation (*n* = 7)^(14–18,20,21)^ or in conjunction with other social media platforms (*n* = 1)^(22)^. Bonnevie *et al*.^(22)^ delivered a digital campaign (‘*Strong Beautiful Future*’) for 2 years that focused on providing pregnancy-related health information via four social media platforms (i.e. Facebook, Instagram, Twitter and YouTube) and a website. Vander Wyst *et al.* ^(20)^ also communicated pregnancy-related health information. However, this was provided via Facebook messages for 18 weeks. Neither of these studies included a control group. The other six studies^(14–18,21)^ created private Facebook groups that could only be accessed by participants in the intervention group. Half of these studies provided information relating to postpartum health for 8 weeks^(14)^, 6 months^(15)^ or 11 months^(16)^. Two studies^(17,18)^ used these groups for 8 weeks to promote physical activity. One study^(21)^ provided health information to pregnant women up until weeks 36–38 of their pregnancy. All six studies included a control group. The final study^(19)^ utilised WeChat to deliver an 8-week mindfulness intervention to pregnant women with mild to moderate symptoms of anxiety or depression. Results were compared with a control group.

### Outcomes and measures

Seven studies^(14,16,17,19–22)^ used self-reported questionnaires to collect outcome data pre- and post-intervention^(14,17,19,20)^ or at three^(21,22)^ to six different time points^(16)^. To assess physical activity, two studies^(17,18)^ used a pedometer, accelerometer and diaries, or solely a pedometer, before, during or after the intervention. Three studies^(17,18,20)^ collected anthropometric measurements, such as weight, height and waist circumference. One study^(20)^ also conducted interviews with the participants pre- and post-intervention, while another^(15)^ utilised monthly phone calls to collect data.

### Studies with a control group

#### Antenatal health

The results of the included studies are presented in Table 2. Two studies^(19,21)^ evaluated pregnancy-related health outcomes and behaviours in both an intervention and control group using self-report questionnaires. In one of these studies^(21)^, both the control and *Expectant Mother Club* intervention group experienced significant improvements in pregnancy adaptation at Time 3 and maternal-fetal attachment at Times 2 and 3 when compared with pre-intervention. There were no differences in outcomes observed between groups.

**Table 2:** Results of the included studies

Author	Changes in health outcomes and behaviours	Social media engagement and acceptability assessment method findings	
Bonnevie^(22)^	• ↑ awareness that regular check-ins with a Dr can lead to healthier babies (66·7 % BL; 80·1 % Yr1; 81·2 % Yr2) • ↑ agreement that women who do not have quality PN care are more likely to have babies born with LBW (46·7 % BL; 60·7 % Yr1; 58·8 % Yr2) • ↑ intentions to talk with a Dr/PN care provider about their baby’s Wt in those that were pregnant/intending to become pregnant (50·0 % BL; 45·5 % Yr1; 68·2 % Yr2) • ↑ awareness of the safe amount of Wt gain during pregnancy (63·6 % BL; 68·8 % Yr1; 75·0 % Yr2) • ↑ awareness that exercising during pregnancy is not dangerous for the baby (56·7 % BL; 63·9 % Yr1; 61·2 % Yr2) • ↑ knowledge of the impacts of LBW into adulthood (23·3 % BL; 42·6 % Yr1; 37·6 % Yr2) • ↑ agreement that health advice for 1 woman may not apply to another (63·3 % BL; 70·5 % Yr1; 76·5 % Yr2)	Metrics were collected using Google Analytics to assess engagement	• The campaign had a total of 1784 followers, with the most on Facebook (920), followed by Instagram (709) and Twitter (155) • During Yr1 and Yr2, Facebook showed the highest avg mthly impressions (31 392 Yr1; 23 234 Yr2), followed by Instagram (4500 Yr1; 11 566 Yr2) • For avg mthly engagements, Instagram showed the highest level of engagements (476 Yr1; 678 Yr2), followed by Facebook (34 Yr1; 438 Yr2) and Twitter (29 Yr1; 104 Yr2) • All social media metrics ↑ from Yr1 to Yr2, except for avg mthly Facebook impressions and avg daily Facebook reach • Videos posted on YouTube during Yr2 received 6772 avg mthly impressions, and 2520 engagements (videos were only available in Yr2) • Website metrics ↑ in the total number of users, avg number of users/mth and total sessions
Boyd^(14)^	• Greater ↑ in parenting competence in IG (67·8 to 76·3), whereas it ↓ in CG (78·3 to 73·6)* • Greater ↓ in depression symptoms in IG (29·5 to 20·2) compared with CG (23·4 to 23·3)*	The proportion of mothers who viewed content in the Facebook group (IG) or attended an in-person session (CG) was used to assess engagement A 5-point Likert scale was used to assess the acceptability of the wkly sessions and the overall intervention (IG only)	• All mothers (100·0 %) in IG attended at least 1 session, whereas 25·0 % of mothers in CG attended at least 1 session • Avg attendance in IG was 83·0 % (compared with 3·0 % in CG), and avg participant commenting was 73·0 % • M score for individual sessions ranged from 3·6 to 4·4 • M satisfaction score for the overall intervention was 4·54
Cavalcanti^(15)^	• IG had higher percentages of EBF each mth compared with CG • EBF percentages ↓ each mth in both groups, and at 6 mths, the smallest percentages of EBF were observed – 33·3 % IG and 8·3 % CG* • Mdn EBF duration was 63 d longer in IG than CG – 149 d *v*. 86 d* • Survival curves of the duration of EBF demonstrated a sharp ↓ in CG compared with IG. The proportional hazard of the early interruption of EBF had a hazard ratio of 0·38*, demonstrating that the intervention was a protective factor that ↓ the early interruption rates of EBF by 62·0 % during the first 6 mths	N/R	N/R
Fiks^(16)^	• At 6 mths, IG was less likely to feed their infant cereal in the bottle compared with CG* • At 9 mths, IG had higher IFSQ healthy feeding behaviour scores compared with CG* • At 9 mths, IG was less likely to pressure their child to finish food compared with CG* • At 6 and 9 mths, there were no differences between groups in infant feeding beliefs on the IFSQ or the timing of solid food introduction • No significant differences between groups for measures of infant sleep • Infants in both groups had similar amounts of screen time and household television use • The groups did not differ in infants’ age at initiation of regular ‘tummy time’ • Other measures of maternal well-being did not differ significantly between the groups	The number of Facebook posts/comments per woman and group was used to assess engagement (IG only) A Likert scale and open-ended questions at infant ages 2, 6 and 9 mths were used to assess acceptability (IG only)	• The women posted a M of 30 times per group/wk • Individual engagement varied by Facebook group – Group 1: Mdn 2·3 posts or comments per individual/wk, Group 2: 2·3, Group 3: 1·5 and Group 4: 3·6 • The women frequently discussed curriculum topics, both in response to prompts and independently initiated. This included conversations about feeding (63 conversations), maternal wellbeing (51), infant activity (40) and infant sleep (29) • >99·0 % of posts centred on infant and parenting topics • During the PN curriculum (first 7 wks), there were 1953 participant posts across the 4 Facebook groups, then 1802 from 0–3 mths PP, 1074 from 3–6 mths PP and 553 from 6–9 mths PP • Individual participation was inversely associated with Wt-for-length *Z*-score*, but not for other parent or child characteristics • Participation in IG did not translate into higher scores on the MSPSS • When asked “*What do you think of the peer group*?” all responses were positive, with most describing the group as “*helpful*” or “*fun*” • 88·0 % responded “*agree*” or “*strongly agree*” to the statements, “*I would recommend this program*” and “*The program was helpful*” • The women appreciated the peer-group approach (“*I think it’s helpful because the advice is coming from other mothers like myself*”) • When asked about the group facilitator, responses were consistently positive (“*she is a big help and very involved and supportive. I love it*”) • When asked, “*What could be improved about the group?*” 60 % responded “*nothing*” while 24·0 % suggested additional in-person meetings
Joseph^(17)^	• 28 women (97·0 %) provided valid accelerometer wear time data • Avg. accelerometer wear time was 786·3 mins/d at BL and 840·2 mins/d at follow-up • No significant BL to 8 wk changes for any of the accelerometer-measured PA outcomes for either group • Between-group differences for changes in sedentary behaviour*, light PA* and moderate PA* • IG ↓ sedentary behaviour by 71 mins/wk, whereas CG ↑ sedentary behaviour by 118 mins/wk* • ↑ in light PA in both IG and CG (95 mins/wk *v*. 59 mins/wk, respectively), with IG demonstrating a greater ↑* • IG ↑ moderate PA by 27 mins/wk, while CG ↓ moderate PA by 35 mins/wk* • EVS data showed that IG ↑ moderate-to-vigorous PA by a Mdn of 50 mins from BL to 8 wks*, while moderate-to-vigorous PA in CG remained relatively stable • ↑ in PA among the IG was greater than CG* • CG performed a higher number of steps. The avg. number of steps/d for both groups remained relatively stable during the first 6 wks and declined during the last 2 wks • Steps/d ↓ in IG* and there was no change in CG • IG reported ↑ in self-regulation for PA* and social support from family for PA*. The only significant ↑ reported by CG was for self-regulation for PA* • ↑ in self-regulation for PA and social support from family for PA among IG was greater than CG* • Between-group difference for changes in outcome expectations for PA favoured CG* • BMI among all women in both groups remained stable	A satisfaction survey was used to assess acceptability of the content of the promotion materials delivered via Facebook/text msg (IG) or mail (CG), as well as the technology-based platforms used to deliver the intervention materials (IG only) Fidelity of intervention delivery was evaluated by several indices – (1) researchers kept logbooks to document the delivery of intervention materials; (2) receipt of the Facebook materials was evaluated by analytic tracking software provided by Facebook; and (3) receipt of text msgs was evaluated by the number of msgs that were returned as “undeliverable” and self-reported receipt of the text msgs (IG only)	• All women (100·0 %) in IG reported gaining PA knowledge from the promotion materials, compared with only 53·0 % in CG • 79·0 % of IG reported that the PA materials were “*helpful*” to “*very helpful*” for promoting PA, 86·0 % reported that were “*satisfied*” or “*very satisfied*” with the program, and all (100·0 %) reported that they would recommend the program to a friend • 26·6 % of CG reported that the promotion materials were “*helpful*” for promoting PA, 47·0 % were “*satisfied*” or “*very satisfied*” with the program and 87·0 % indicated that they would recommend the program to a friend • 93·0 % of IG reported gaining PA knowledge from the text msgs and 79·0 % indicated they were “*helpful*” to “*very helpful*” in promoting PA • 94·0 % of IG reported that they were “*motivated*” or “*very motivated*” to continue being physically active after the study, compared with 7·0 % of CG • Written qualitative feedback supported the intervention program, e.g., “*It provided a sense of accountability and it was good to hear what other participants were saying*”, “*The program was good and I think it will be helpful to others*”, “*Loved the information uploaded on the Facebook page regarding PA*”, and “*Repeat the study, it is very much needed in my community*” • All 8 of the wkly Facebook posts and discussion topics were successfully delivered as scheduled • Analytic tracking software indicated that all women (100·0 %) viewed the wkly PA promotion posts and group discussion topics during the first half of the study (wks 1–4); however, during the latter half of the study, the number of women who viewed the wkly PA promotional posts and group discussion topics declined (64·0–86·0 %) • 50·0 % of the women viewed all the PA promotion materials posted on the group wall and the Mdn number of comments for each wkly group discussion topic was 5·5
Rote^(18)^	• Avg ↑ in steps/d in both groups*, however there was a greater ↑ in IG* (5295·22 to 12 472·44 IG and 5595·10 to 10 135·64 CG) • Inspection of the M indicated that IG began to diverge from CG between wks 1 and 2, with the difference becoming larger after wk 4 • Simple main effect comparisons of both groups at each time point, however, were significant only at wks 7 and 8* • WC ↓ by 1·1 cm in IG* (no significant changes in CG) • Only significant predictor of change in steps/d for IG was BL steps/d • BL steps/d was a significant predictor of ↑ in steps/d for CG • For both groups, the lower the BL PA level, the greater ↑ in steps/d	The number and type of posts made by the women were used to assess engagement (IG only)	• Levels of engagement ↓ throughout the intervention. This factor was not a significant predictor of change in PA • Adherence to the intervention did not predict change in steps/d • Avg number of posts per woman was just over 7 • The avg number of posts/d to the groups was 1 • Although all the women engaged in their Facebook group, there was a wide range of engagement with a minimum of 6 posts to a maximum of 32 posts
Vander Wyst^(20)^	• No woman gained less than the recommended amount of Wt. However, nearly 67·0 % of the women gained excessive Wt during gestation • M kcal consumption ↓ slightly from 2336·75 to 2323·67 kcal • M Pr intake was within the AMDR at BL (14·4 %) and post-intervention (15·6 %) • M CHO intake was within the AMDR at BL (54·6 %) and post-intervention (57·8 %) • M fat intake was above the AMDR at BL (37·6 %) and post-intervention (35·1 %) • Sugar consumption ↑ from BL to post-intervention from 125·2 g to 131·5 g* • Women who consumed fast food < 1/wk ↓ from 54·5 % at BL to 44·4 % post-intervention • Breakfast was skipped most often compared with other meals, with 18·0 % of women skipping breakfast at BL and 33·0 % post-intervention • Common themes from the interviews were dietary changes (18·2 %), PA (12·1 %) and using the internet as a source for pregnancy-related health information (10·9 %) • 69·0 % of the women reported making dietary changes for the health of their baby • The women reported exercising during pregnancy not only for the health of themselves and their babies but also to help with PP Wt loss	The number of msgs that were viewed (passive) and liked (active) was used to assess passive and active engagement. Active participation was also determined by recording individual responses to poll questions that were sent via Facebook or text msg. Bitly (https://bitly.com) short links to health websites and videos were included in the msgs and the number of clicks on each link was tracked Follow-up interviews were conducted with each woman to assess acceptability	• Of the 65 Facebook msgs that each woman received, a Mdn of 60 msgs were viewed • A total of 49 clicks were recorded across all 10 msg categories. Links with the most interaction contained PA and Wt gain msgs. Other categories of interest were healthy snacks, recipes and relaxation/music • All women (100·0 %) reported finding the msgs they received helpful • Dietary suggestions and recipes, in particular, were well-received, e.g., 1 woman reported adding milk to her coffee after reading a msg about the importance of milk. Other msgs about comfortable sleeping and health reminders were also enjoyed • While many msgs related to the health benefits of breast-feeding, this was only mentioned by 3 women during the interviews • The women responded well to the format of the msgs and enjoyed the friendly, relaxed tone
Wu^(21)^	• No significant differences in the interaction effects of group and time in all main outcomes. However, time effects on the maternal–fetal attachment at Time 2 and 3 and pregnancy adaptation at Time 3 were determined in both groups (combined) compared with Time 1 • Compared with Time 1, the M scores on maternal–fetal attachment ↑ at Time 2* and Time 3* (groups combined) • Compared with Time 1, the M scores of pregnancy adaptation ↑ at Time 3* (groups combined)	A survey regarding virtual community preferences was completed at Time 3 and was used to assess acceptability (IG only)	• Women were categorised by their time spent on the *EMC* into ‘high-adherence’ (≥150 min/mth) and ‘low-adherence’ groups (<150 min/mth). Fourteen women were classified as ‘high-adherence’ and 45 as ‘low-adherence’ • Most women visited the *EMC* at night (6 pm–12 am) • Women in the ‘high-adherence’ group often used their computers (57·1 %) as access devices, and those in the ‘low-adherence’ group used smartphones (60·0 %) more often • Women in the ‘high-adherence’ group reported higher scores on the activity preferences for discussions with others, sharing ultrasound pictures with others and reading pregnancy information* than the ‘low-adherence’ group • ‘High-adherence group’ reported higher satisfaction with library information* • First-time pregnant women and women whose pregnancy was assisted by a technology treatment had higher adherence to the virtual community*
Yang^(19)^	• M number of mindfulness meditations/wk was 3·25 • M number of mins spent on meditation each time was 21·23 mins • All women (100·0 %) learned the skills of attention monitoring, but it was more difficult to learn the ability to tolerate discomfort and adopt an accepting attitude towards all experiences • The M scores for IG of the PHQ-9 and GAD-7 pre-intervention were 5·98 and 5·52, respectively, indicating mild symptoms of depression and anxiety. These scores ↓ to 3·58* (PHQ-9) and 2·97* (GAD-7) post-intervention, indicating no symptoms • No changes in the PHQ-9 and GAD-7 scores were observed in CG • Post-intervention scores of the PHQ-9 and GAD-7 were lower in IG* • A larger proportion of IG had no symptoms of depression or anxiety post-intervention (41 and 49 women, respectively) compared with CG (22 and 26 women, respectively) • No differences in FFMQ scores or scores for any of the subscales were observed between the groups at BL • FFMQ scores and scores for the observing subscale (corresponding to attention monitoring) and the non-judgement and non-reactivity subscales (corresponding to acceptance) ↑ in IG (M score ↑ of 7·47*, 2·18*, 2·05* and 2·99*, respectively), but there were no changes in the scores for subscales in CG	Self-report login data, login records and post-intervention surveys were used to assess engagement and acceptability (IG only)	• 83·9 % of women completed at least 3 sessions • 86·5 % reported that the mindfulness intervention was beneficial • 53·8 % felt relaxed and calm during the mindfulness meditation • 40·4 % found that the mindfulness practice helped them become fully aware of fetal movement • 23·1 % reported that the intervention relieved discomfort and allowed them to be more energetic • Adherence to daily mindfulness practice was low • Some women reported that learning to maintain an accepting attitude was challenging

AMDR, acceptable macronutrient distribution range; Avg, average; BL, baseline; CG, control group; CHO, carbohydrate; Dr, doctor; EBF, exclusive breast-feeding; EMC, Expectant Mother Club; EVS, exercise vital sign questionnaire; FFMQ, five facets of mindfulness questionnaire; g, gram; GAD-7, generalised anxiety disorder scale; IFSQ, infant feeding style questionnaire; IG, intervention group; kcal, kilocalorie; LBW, low birth weight; M, mean; Mdn, median; Min, minute; Msg, message; MSPSS, maternal scale of perceived social support; Mth, month; N/A, not applicable; N/R, not reported; PA, physical activity; PHQ-9, The Patient Health Questionnaire; PN, prenatal; PP, postpartum; Pr, protein; WC, waist circumference; Wk, week; Wt, weight; Yr, year.

* Indicates significance.

In the other study, while adherence to daily mindfulness practice was relatively low, Yang *et al.* ^(19)^ found significant improvements in depression and anxiety symptoms, as well as an increase in mindfulness skills, in the intervention group post-intervention. Conversely, changes in depression and anxiety symptoms post-intervention were not significant for the control group. Furthermore, a greater number of women in the intervention group had no symptoms of depression or anxiety when compared with the control group post-intervention. Lastly, for the Five Facets of Mindfulness Questionnaire, the overall scores, the scores for the observing subscale (corresponding to attention monitoring) and the scores for the non-judgement and non-reactivity subscales (corresponding to acceptance) significantly improved for the intervention group but not the control group.

#### Postpartum health

Three studies^(14–16)^ evaluated health outcomes or behaviours in postpartum women using self-report questionnaires^(14,16)^ or by contacting participants via phone^(15)^. Boyd *et al.* ^(14)^ observed a greater reduction in depression symptoms in the intervention group compared with the control group post-intervention. Additionally, parenting competence scores increased in the intervention group and decreased in the control group.

In addition to maternal health outcomes, infant feeding practices were also impacted by postpartum interventions. The second study^(16)^ identified significantly healthier feeding practices in the intervention group. These mothers were less likely to feed their children cereal in their bottles at 6 months and pressure their children into finishing their food at 9 months. Post-intervention, scores for the Infant Feeding Style Questionnaire were higher in the intervention group. However, infant feeding beliefs, the timing of solid food introduction, infant sleep, screen time, household television use, the timing of the initiation of regular ‘tummy time’ and other measures of maternal well-being did not differ between the groups.

Cavalcanti *et al*.^(15)^ found a significant difference in the median exclusive breast-feeding duration, with a difference of 63 d between the intervention and control groups. At the 6-month follow-up, one-third of participants in the intervention group were still breast-feeding, compared with under 10 % in the control group indicating that participation in the intervention reduced early interruption of breast-feeding in the first 6 months by 62 %.

#### Physical activity

Two studies assessed physical activity outcomes in either adult^(17)^ or college-aged women^(18)^ in an intervention and a control group. Using a range of self-reported (questionnaires and calendars) and objective (accelerometers and pedometers) data, Joseph *et al*.^(17)^ found significant between-group differences for sedentary behaviour, light physical activity and moderate physical activity from pre- to post-intervention. Sedentary behaviour decreased in the intervention group and increased in the control group. While light physical activity increased in both groups, a significantly greater increase occurred in the intervention group. Moderate physical activity increased in the intervention group and decreased in the control group. No significant changes in the data captured by the accelerometers were found for either group. For both groups, the number of self-reported steps per day remained stable until week 6 and then declined until the end of the intervention, significantly for the intervention group only.

Results of the questionnaires demonstrated a significant increase in moderate-to-vigorous physical activity in the intervention group and stable results in the control group. In addition, these data showed significantly greater increases in physical activity, self-regulation for physical activity and social support from family in the intervention group. Self-regulation for physical activity was the only significant increase found in the control group and the significant between-group difference for changes in outcome expectations for physical activity favoured the control group. BMI remained stable for all participants from pre- to post-intervention.

Rote *et al*.^(18)^ investigated changes in steps per day and anthropometric measurements. Although both groups achieved significant increases in the number of steps per day, a more significant increase occurred in the intervention group. In both groups, a lower baseline activity level and baseline steps per day predicted a greater increase in steps per day.

Regarding anthropometric measurements, a significant decrease in waist circumference was observed in the intervention group. However, there were no significant changes in the control group.

### Studies without a control group

#### Prenatal health

Bonnevie *et al*.^(22)^ evaluated prenatal health outcomes and behaviours in only an intervention group. Questionnaire results demonstrated that their campaign had a positive impact on the target population. For example, over time, there was increased: (1) awareness that regular check-ins with a doctor can lead to healthier babies; (2) awareness of the safe amount of weight gain during pregnancy and (3) knowledge of the impacts of low birth weight into adulthood. However, due to the small sample size of this study, none of the results achieved statistical significance.

#### Antenatal health

Vander Wyst *et al*.^(20)^ assessed antenatal health outcomes and behaviours in only an intervention group using both self-reported (questionnaires and interviews) and objective (hospital delivery weight) data. Regarding diet quality, mean caloric consumption did not significantly change. Protein and carbohydrate intakes were within the acceptable macronutrient distribution range at baseline and post-intervention. Conversely, pre-intervention fat intake was higher than the acceptable macronutrient distribution range and although it reduced post-intervention, it remained slightly above the acceptable macronutrient distribution range. Compared with other main meals, breakfast was skipped most often. None of the women gained less than the recommended amount of weight; however, around two-thirds gained excessive weight during their pregnancy. In their interviews, the women mentioned making dietary changes for the health of their babies and exercising during pregnancy, not only for the health of themselves and their babies but also to help with postpartum weight loss. Furthermore, although this intervention was well-received by the women, it did not lead to large changes in dietary quality or knowledge.

### Social media engagement and acceptability

Eight of the included studies^(14,16–22)^ evaluated social media engagement, defined as any measure of social media intervention use, or acceptability, defined as any measure of participant satisfaction with the intervention (Table 2). These measures were assessed using a variety of methods, including self-report questionnaires, interviews with participants and activity in the Facebook groups.

#### Social media engagement

Social media engagement was examined by seven studies^(14,16,18–22)^. Bonnevie *et al*.^(22)^ used Google Analytics to collect metrics and assess digital engagement with their social media campaign. Engagement remained strong throughout the 2-year intervention period. Aside from average monthly Facebook impressions and average daily Facebook reach, all other digital metrics increased between Years 1 and 2. Notably, Instagram had the highest level of engagements (i.e. likes, comments, shares, video views or post clicks) with 476 in Year 1 and 678 in Year 2. This was followed by Facebook and then Twitter.

Boyd *et al*.^(14)^ used the number of participants who viewed the weekly content posted in the ‘*Parents Interacting with Infants’* Facebook group to assess engagement. All mothers in the intervention group viewed at least one of the weekly posts, whereas only one-quarter of mothers in the control group attended at least one in-person session. Average attendance by the intervention group was also considerably higher (83 % *v*. 3 %).

Fiks *et al*.^(16)^ evaluated engagement by tracking the number of Facebook posts and comments in the ‘*Grow2Gether’* intervention. Both the mothers and the facilitators posted about the curriculum topics, with almost all posts (99 %) focusing on infant and parenting topics. Overall, the most frequently discussed topics were feeding (63 conversations) and maternal well-being (51 conversations). The number of Facebook posts gradually decreased throughout the intervention period from 1953 during the first 7 weeks (prenatal period) to 553 when the infants were aged between 6 and 9 months. This study also concluded that individual participation was only inversely associated with infant weight-for-length *Z*-score and participation in the intervention group did not result in higher scores on the Maternal Scale of Perceived Social Support questionnaire.

Rote *et al*.^(18)^ determined engagement in their Facebook Social Support Group by tracking the number of times the participants posted in this group and their interaction with the intervention materials. Engagement levels varied with participants interacting with between six and thirty-two posts. In addition, engagement decreased throughout the intervention. However, this was not a significant predictor of change in physical activity, nor did adherence to the intervention predict changes in the number of steps per day.

Vander Wyst *et al.* ^(20)^ measured passive engagement with Facebook messages by tracking how many messages each participant viewed. In addition, URL to health websites and videos were included in the messages and active participation was assessed by tracking the number of clicks on each link. A median of 92 % of the messages were viewed by the participants and links relating to exercise and weight gain were clicked on the most. Links directing the mothers to healthy snacks, recipes and relaxation/music were also of particular interest.

After the intervention, Wu and Hung^(21)^ utilised a self-report questionnaire to collect information regarding the participant’s virtual community use preferences. Most participants (76 %) were classified as ‘low-adherence’, as they spent less than 150 min/month on the Expectant Mother Club. Mothers in the ‘high-adherence’ group reported significantly higher satisfaction with the library information. Lastly, higher adherence to the Expectant Mother Club was observed in first-time mothers and women whose pregnancy was assisted by a technology treatment.

Yang *et al*.^(19)^ used self-report and login data from the WeChat application to determine engagement. While most women (84 %) completed at least three mindfulness meditations, adherence to daily mindfulness practice was relatively low. On average, the women completed 3·25 mindfulness meditations per week.

#### Social media acceptability

Five studies^(14,16,17,19,20)^ investigated the acceptability of social media interventions. Boyd *et al.* ^(14)^ used a 5-point Likert scale (with 1 = low and 5 = high) to assess satisfaction with the weekly sessions and the overall intervention. Favourable scores were observed for both measures, with mean scores ≥3·6.

Similarly, Fiks *et al*.^(16)^ used a Likert scale, as well as open-ended questions, to evaluate acceptability when the infants were aged 2, 6 and 9 months. Positive feedback was received from most participants. For example, most mothers described the *‘Grow2Gether’* Facebook group as ‘helpful’ or ‘fun’ in response to ‘What do you think of the peer group?’, and 88 % answered ‘agree’ or ‘strongly agree’ when asked ‘I would recommend this program’ and ‘the program was helpful’.

A 26-item questionnaire was utilised by Joseph *et al*.^(17)^ to determine the acceptability of the content of the physical activity promotion materials/text messages and the platforms used to deliver the intervention. This was compared with a 19-item satisfaction survey completed by the control group. Results from these surveys supported the intervention program. A greater proportion of participants in the intervention group gained physical activity knowledge (100 % *v*. 53 %) and found the intervention ‘helpful’ or ‘very helpful’ in promoting physical activity (79 % *v*. 27 %). The physical activity promotion text messages were also well-received by the intervention group, with most participants gaining physical activity knowledge (93 %) and finding them ‘helpful’ or ‘very helpful’ (79 %). Additionally, the intervention participants rated the overall intervention higher, with almost double the number of participants reporting they were ‘satisfied’ or ‘very satisfied’ with the intervention compared with the control group (86 % *v*. 47 %).

Vander Wyst *et al.* ^(20)^ conducted follow-up interviews with each participant to gain feedback on their Facebook message intervention. During these interviews, all mothers reported finding the messages helpful. In particular, messages relating to dietary suggestions, recipes, comfortable sleep and health reminders were well-received.

To obtain feedback on their mindfulness intervention, Yang *et al.* ^(19)^ sent the participants a self-report questionnaire after the final session. Most (87 %) found the intervention to be beneficial and all mothers learnt attention monitoring skills. However, it was more challenging to learn the ability to tolerate discomfort, adopt an accepting attitude towards all experiences and maintain an accepting attitude.

### Risk of bias

Three randomised control trials were judged as low risk of bias^(14,15,19)^ and three as moderate risk^(16–18)^ (online Supplemental Table 2(a)). The cross-sectional study was judged as moderate risk^(22)^ (online Supplemental Table 2(b)). Both quasi-experimental studies were judged as low risk^(20,21)^ (online Supplemental Table 2(c)).

## Discussion

This is the first review to examine the nature, effectiveness and acceptability of social media-delivered health promotion interventions in adult women of reproductive age. The interventions were diverse and differed in terms of their comparator groups, duration and data collection methods. Despite this diversity, our results indicate that social media is a potentially useful and acceptable avenue for improving a range of health outcomes and behaviours in adult women of reproductive age. In the nine studies identified, a range of health outcomes and behaviours were targeted, including diet quality, breast-feeding duration, physical activity and mental health, in mothers, expectant mothers and women of reproductive age.

### Social media-delivered health promotion interventions

In this review, the most used social media platform was Facebook, with six studies^(14–18,21)^ creating a private Facebook group, one study^(20)^ sending health information via Facebook messages and one study^(22)^ delivering part of their social media campaign via Facebook. Only one study^(22)^ used more than one social media platform. Results from this study indicated that Instagram had the highest level of monthly engagements compared with Facebook, Twitter and YouTube. This finding is consistent with current data, which demonstrates that a higher percentage of Instagram’s global users are women of reproductive age^(3,4)^. The social media landscape is constantly changing and evolving. Therefore, future research should consider which social media platform their target audience engages with the most to deliver effective interventions.

Aside from the platform used for intervention delivery, our review highlighted a high degree of diversity among interventions, including their use of comparator groups, duration, data collection methods and outcomes assessed. Similarly, this diversity has been reported by other systematic reviews, including a review examining the effect of interactive social media interventions on health outcomes in the general adult population^(5)^, another assessing computer- or web-based interventions in perinatal mental health^(6)^ and another assessing the influence of social networking sites on health behaviour change^(23)^. These findings highlight the need for greater consistency across future studies, including outcomes assessed and targeted populations, to identify intervention components that are most effective in women of reproductive age.

### Changes in health outcomes and behaviours

All the included studies reported positive changes in health outcomes and behaviours from baseline to post-intervention. However, not all changes achieved statistical significance. Findings from the five studies^(14–18)^ that focused on either postpartum health or physical activity were primarily positive and significant differences were observed between the intervention and control groups including a greater reduction in depression severity and increased parenting competence^(14)^, longer duration of breast-feeding^(15)^, healthier feeding practices^(16)^ and increased physical activity^(17,18)^. However, in studies that focused on prenatal or antenatal health^(19–22)^ results were mixed with few significant findings. As prenatal and antenatal care traditionally involves regular contact with a healthcare professional (e.g. midwife, obstetrician or general practitioner)^(24)^, the impact of these additional social media interventions may have been reduced. Conversely, the frequency of this contact decreases postnatally. Therefore, as women may not be receiving as much health information or support, they may be more likely to engage with and benefit from social media interventions during the postpartum period.

Similar results were found in a 2021 systematic review^(5)^ that examined the effect of interactive social media interventions on health outcomes in the general adult population (i.e. males and females aged > 18 years). While not specific to adult women of reproductive age, six of the nine studies included in our review were also included in this 2021 systematic review. Both reviews found evidence that social media interventions can improve a range of health outcomes and behaviours.

### Social media acceptability and engagement

Our findings suggest that social media interventions have higher rates of acceptability and engagement when compared with traditional in-person interventions and standard care procedures. Most noticeably, Boyd *et al.* ^(14)^ observed an average attendance of 83 % in the group receiving the Parents Interacting with Infants parenting intervention via Facebook compared with only 3 % in the group attending in-person sessions. With traditional in-person interventions, only participants who can travel to the specified location at a fixed time will receive the intervention^(25)^. This can be a significant challenge for women of reproductive age, especially mothers and expectant mothers with competing priorities. Social media interventions address this limitation by allowing women to participate in an intervention at a time and place convenient to them. Moreover, given women are highly engaged users of social media, and these platforms are easily accessible and of a low cost, the use of social media in health promotion interventions for this population has great potential.

### Strengths and limitations

This review was conducted in accordance with PRISMA-ScR guidelines. A systematic search in six databases combined with comprehensive hand searching was implemented to capture relevant records. While the search was completed by one author, the screening process, quality assessment and data extraction were completed in duplicate.

However, the results of this review should also be interpreted in the context of the following limitations. First, our understanding of the long-term effectiveness of health promotion interventions conducted via social media in adult women of reproductive age is limited. Most studies (*n* = 6) were conducted for less than 6 months^(14,15,17–20)^, and no studies provided long-term follow-up to assess if changes in health outcomes and behaviours were maintained beyond the intervention period. While no studies reported adverse events in the short term, it is unclear as to whether this would also be the case if the interventions were implemented for an extended period. Moreover, further research is required to ascertain the long-term efficacy of social media interventions.

It is also important to consider that most studies (*n* = 8)^(14–17,19–22)^ used self-report data to assess changes in health outcomes and behaviours. While not the primary mode of delivery, several studies included additional intervention components, including information booklets and text messaging. These components may have contributed to the overall intervention effect; therefore, findings may not be attributed solely to the social media-delivered aspect of the intervention.

Several factors restrict the generalisability of our results. As this is an emerging area of research, a limited number of studies were included in the present review. Most of these studies had a small sample size. Therefore, it is possible that the included studies were subject to small-study effects, whereby smaller sample sizes show larger and more favourable intervention effects^(27)^. In addition, of the six studies^(14,16–18,20,22)^ that provided information on the ethnicity of their participants, four^(14,16,17,22)^ included mainly African American/Black subjects from developed countries. Given the practicality of health interventions in adult women varies according to a range of factors, including ethnic and economic^(28)^, results may not be generalisable to all females of reproductive age. This highlights the need for additional studies with adequate sample sizes and diverse populations, including women of varying ethnicities and at different life stages.

Lastly, most studies were judged as having a low to moderate risk of bias. This, in addition to the small study samples, short intervention durations and the absence of comparator groups in some studies, may affect the certainty of our evidence.

### Conclusion

To date, there have been limited health promotion intervention studies delivered via social media to women of reproductive age, with high heterogeneity in all aspects of studies. Based on the available evidence, health promotion interventions conducted via social media appear to be acceptable and effective for improving a variety of health outcomes and behaviours in adult women of reproductive age. Our findings suggest that health promotion via social media may be ideal for this population, and deliverable, at scale. However, further research is required to prove its effectiveness and superiority over other intervention strategies.

## Supporting information

Henderson and Gow supplementary material Henderson and Gow supplementary material

## Supplementary material

For supplementary material accompanying this paper visit https://doi.org/10.1017/S136898002300246X
