# Supplementary material for: A scoping review of health promotion interventions delivered via social media to women of reproductive age
Source: Public Health Nutr. 2023 Nov 6;26(12):3173–89. doi: 10.1017/S136898002300246X (PMC10755387; doi:10.1017/S136898002300246X)
Supplement: Henderson and Gow supplementary material [file S136898002300246Xsup001.docx]

**Supplementary materials**

A scoping review of health promotion interventions delivered via social media to women of reproductive age

**Supplementary Table 1.** Example search strategy

**Supplementary Table 2A.** Randomised control trials quality assessment

**Supplementary Table 2B.** Cross-sectional studies quality assessment

**Supplementary Table 2C.** Quasi-experimental studies quality assessment

**Supplementary Table 1.** Example search strategy

| **MEDLINE via Ovid (1946-present)**   \| 1. Postpartum.mp. or exp Postpartum Period/ \| \| --- \| \| 1. Postpartum Women.mp. \| \| 1. Postnatal.mp. \| \| 1. Puerper*.mp. \| \| 1. Perinatal.mp. \| \| 1. Pregnant Women.mp. or exp Pregnant Women/ \| \| 1. Pregnan*.mp. \| \| 1. OR 1-7 \| \| 1. Health Promotion.mp. or exp Health Promotion/ \| \| 1. Health Interventio*.mp. \| \| 1. Health Promotion Campaign.mp. \| \| 1. OR 9-11 \| \| 1. Social Media.mp. or exp Social Media/ \| \| 1. Internet-based Intervention.mp. or exp Internet-based Intervention/ \| \| 1. Social Networ*.mp. \| \| 1. Online.mp. \| \| 1. Internet.mp. \| \| 1. Facebook.mp. \| \| 1. Instagram.mp. \| \| 1. OR 13-19 \| \| 1. Mental Health.mp. or exp Mental Health/ \| \| 1. Depression.mp. \| \| 1. Anxiety.mp. \| \| 1. Exercise.mp. or exp Exercise/ \| \| 1. Physical Activit*.mp. \| \| 1. Diet.mp. or exp Diet/ \| \| 1. OR 21-26 \| \| 1. AND 8, 12, 20, 27 \| |
| --- | --- | --- | --- | --- | --- | --- | --- | --- | --- | --- | --- | --- | --- | --- | --- | --- | --- | --- | --- | --- | --- | --- | --- | --- | --- | --- | --- | --- |

**Supplementary Table 2A.** Randomised control trials quality assessment

| **Author** | **Q1** | **Q2** | **Q3** | **Q4** | **Q5** | **Q6** | **Q7** | **Q8** | **Q9** | **Q10** | **Q11** | **Q12** | **Q13** | **% Yes** | **Risk** |
| --- | --- | --- | --- | --- | --- | --- | --- | --- | --- | --- | --- | --- | --- | --- | --- |
| Boyd^(14)^ | Y | Y | N | N | N | N/A | Y | Y | Y | Y | Y | Y | Y | 75 | Low |
| Cavalcanti^(15)^ | Y | Y | Y | Y | N | U | Y | Y | Y | Y | Y | Y | Y | 85 | Low |
| Fiks^(16)^ | Y | Y | N | N | N | U | Y | Y | Y | Y | Y | Y | Y | 69 | Moderate |
| Joseph^(17)^ | Y | U | Y | N | N | U | Y | Y | N | Y | Y | Y | Y | 62 | Moderate |
| Rote^(18)^ | Y | Y | U | U | N | U | Y | U | N | Y | Y | Y | Y | 54 | Moderate |
| Yang^(19)^ | Y | Y | Y | N | N | Y | Y | Y | Y | Y | Y | Y | Y | 85 | Low |
| Q1. Was true randomisation used for assignment of participants to treatment groups?, Q2. Was allocation to treatment groups concealed?, Q3. Were treatment groups similar at the baseline?, Q4. Were participants blind to treatment assignment?, Q5. Were those delivering treatment blind to treatment assignment?, Q6. Were outcomes assessors blind to treatment assignment?, Q7. Were treatment groups treated identically other than the intervention of interest?, Q8. Was follow up complete and if not, were differences between groups in terms of their follow up adequately described and analysed?, Q9. Were participants analysed in the groups to which they were randomised?, Q10. Were outcomes measured in the same way for treatment groups?, Q11. Were outcomes measured in a reliable way?, Q12. Was appropriate statistical analysis used?, Q13. Was the trial design appropriate, and any deviations from the standard RCT design (individual randomization, parallel groups) accounted for in the conduct and analysis of the trial?  Y: yes, N: no, U: unclear, N/A: not applicable (excluded from count) | | | | | | | | | | | | | | | |

**Supplementary Table 2B.** Cross-sectional studies quality assessment

| **Author** | **Q1** | **Q2** | **Q3** | **Q4** | **Q5** | **Q6** | **Q7** | **Q8** | **% Yes** | **Risk** |
| --- | --- | --- | --- | --- | --- | --- | --- | --- | --- | --- |
| Bonnevie^(22)^ | Y | Y | Y | N/A | N | N | Y | N | 57 | Moderate |
| Q1. Were the criteria for inclusion in the sample clearly defined?, Q2. Were the study subjects and the setting described in detail?, Q3. Was the exposure measured in a valid and reliable way?, Q4. Were objective, standard criteria used for measurement of the condition?, Q5. Were confounding factors identified?, Q6. Were strategies to deal with confounding factors stated?, Q7. Were the outcomes measured in a valid and reliable way?, Q8. Was appropriate statistical analysis used?  Y: yes, N: no, U: unclear, N/A: not applicable (excluded from count) | | | | | | | | | | |

**Supplementary Table 2C.** Quasi-experimental studies quality assessment

| **Author** | **Q1** | **Q2** | **Q3** | **Q4** | **Q5** | **Q6** | **Q7** | **Q8** | **Q9** | **% Yes** | **Risk** |
| --- | --- | --- | --- | --- | --- | --- | --- | --- | --- | --- | --- |
| Vander Wyst^(20)^ | Y | Y | Y | N | Y | Y | Y | Y | Y | 89 | Low |
| Wu^(21)^ | Y | Y | Y | Y | Y | Y | Y | Y | Y | 100 | Low |
| Q1. Is it clear in the study what is the ‘cause’ and what is the ‘effect’ (i.e., there is no confusion about which variable comes first)?, Q2. Were the participants included in any comparisons similar?, Q3. Were the participants included in any comparisons receiving similar treatment/care, other than the exposure or intervention of interest?, Q4. Was there a control group?, Q5. Were there multiple measurements of the outcome both pre and post the intervention/exposure?, Q6. Was follow up complete and if not, were differences between groups in terms of their follow up adequately described and analysed?, Q7. Were the outcomes of participants included in any comparisons measured in the same way?, Q8. Were outcomes measured in a reliable way?, Q9. Was appropriate statistical analysis used?  Y: yes, N: no, U: unclear | | | | | | | | | | | |
